# An enhanced recovery after surgery program in orthopedic surgery: a systematic review and meta-analysis

**DOI:** 10.1186/s13018-019-1116-y

**Published:** 2019-03-13

**Authors:** Zhi-Chao Hu, Lin-Jie He, Dong Chen, Xiao-Bin Li, Zhen-Hua Feng, Cheng-Wei Fu, Jiang-Wei Xuan, Wen-Fei Ni, Ai-Min Wu

**Affiliations:** 1 Department of Orthopaedics, The Second Affiliated Hospital and Yuying Children’s Hospital of Wenzhou Medical University, Wenzhou, 325027 Zhejiang China; 2 The Second School of Medicine, Wenzhou Medical University, Wenzhou, 325027 Zhejiang China; 3 Bone Research Institute, The Key Orthopaedic Laboratory of Zhejiang Province, Wenzhou, 325027 Zhejiang China; 4 Department of Acupuncture and Massage, Hubei University of Traditional Chinese Medicine, Wuhan, 430000 Hubei China

**Keywords:** Enhanced recovery after surgery, Orthopedic surgery, Meta-analysis

## Abstract

**Objectives:**

There is an increased interest in enhanced recovery after surgery (ERAS) minimizing adverse events after orthopedic surgery. Little consensus supports the effectiveness of these interventions. The purpose of present systematic review and meta-analysis is to comprehensively analyze and evaluate the significance of ERAS interventions for postoperative outcomes after orthopedic surgery.

**Methods:**

PubMed, EMBASE, and Cochrane databases were totally searched from the inception dates to May 31, 2018. Two reviewers independently extracted the data from the selected articles using a standardized form and assessed the risk of bias. The analysis was performed using STATA 12.0.

**Results:**

A total of 15 published studies fulfilled the requirements of inclusion criteria. We found that the ERAS group showed a significant association with lower incidence of postoperative complications (OR, 0.70; 95% CI, 0.64 to 0.78). Meanwhile, ERAS was also associated with the decline in 30-day mortality rate and Oswestry Disability Index (ODI). However, no significant differences were identified between the two groups regarding the 30-day readmission rate (*P* = 0.397).

**Conclusions:**

Our meta-analysis suggested that the ERAS group had more advantages in reducing incidence of postoperative complications, 30-day mortality rate, and ODI after orthopedic surgery, but not of 30-day readmission rate. However, further research with standardized, unbiased methods and larger sample sizes is required for deeper analysis.

**Electronic supplementary material:**

The online version of this article (10.1186/s13018-019-1116-y) contains supplementary material, which is available to authorized users.

## Introduction

Minimally invasive surgery, damage control surgery, and enhanced recovery after surgery (ERAS) are considered to be the latest three advances in surgery in the twenty-first century [1–3]. However, the real breakthrough in medical science turns out to be fairly difficult. The implementation of ERAS, whose every detail of the perioperative treatment has been taken into account, appears to be the most practical direction of the efforts of surgeons [4–8].

The concept of ERAS was first presented by Danish surgeon Kehlet [9] in the 1990s. Kehlet used evidence-based medical interventions to reduce the stress response of surgical trauma and complications, improve surgical safety and patient satisfaction, so as to achieve the purpose of accelerating rehabilitation. The underlying principle of ERAS is to modulate the surgical stress response to shorten length of stay (LOS), reduce postoperative complication, and achieve faster recovery [10, 11]. Furthermore, its earliest application to gastrointestinal surgery has been proved successful [12–17].

In recent years, the concept of ERAS has reached a consensus in a number of surgical fields and is well established, such as the total hip replacement [18–22]. And it turns out to be credible that the ERAS can make sense in postoperative recovery, which is also a critical part in orthopedics. But there are still few reports and systematic studies on ERAS or fast-track surgery in the specific area of orthopedics. Meanwhile, the effectiveness of ERAS on orthopedics has not been uniformly recognized or accepted by all orthopedic surgeons [23, 24]. Therefore, the purpose of this study is to use meta-analysis to systematically review the outcomes of the ERAS’s application in orthopedics to guide clinical practice.

## Materials and methods

This is a systematic review and meta-analysis study of previous reports, and none of primary personal data will be collected; therefore, the ethical approval is not necessary. And our present systematic review and meta-analysis was performed according to the Preferred Reporting Items for Systematic Review and Meta-Analyses (PRISMA) guidelines [25, 26] (Additional file 1: Checklist).

### Search strategy

#### Databases

Two authors independently searched the electronic literature database of PubMed, EMBASE, and Cochrane databases from the inception dates to May 31, 2018. Related articles and reference lists were searched to avoid original miss. The reference studies of previous systematic reviews, meta-analysis, and included studies were manually searched to avoid initial miss. After the two authors assessed the potentially eligible studies independently, any disagreement was discussed and resolved with the third independent author.

#### Search criteria

Search results were screened by scanning abstracts for the following exclusion criteria: publication of abstracts only, respective studies, case reports, case series, letters, comments, reviews, or meta-analyses; animal studies; duplicate studies; intervention does not meet the inclusion criteria; and lack of detailed data of the outcomes. After removing excluded abstracts, full articles were obtained and studies were screened again more thoroughly using the same exclusion criteria. The key words were used as follows: ERAS, enhanced recovery, fast track, accelerated tracks, spine, THA, TKA, fracture, orthopedics, hip, and knee.

### Study selection

#### Design

We selected studies comparing ERAS interventions with only routine care treatments. And we restricted our meta-analysis to the inclusion criteria which meet following details: prospective and observational retrospective trials, compare the clinical outcomes of ERAS group versus traditional non-ERAS group, and the participants were patients after orthopedic surgery.

#### Outcomes of interest

We screened all identified articles by scanning abstracts or portions of the text to determine if they met the inclusion criteria. Any disagreements were resolved through discussion and consensus between the reviewers. Our primary outcomes were total incidence of postoperative complications. Meanwhile, 30-day mortality rate, 30-day readmission rate, and Oswestry Disability Index (ODI) were our secondary outcomes. We evaluated complication rates including septic shock, myocardial infarction, stroke, cardiac arrest, progressive kidney injury or renal failure, respiratory failure requiring mechanical ventilation, venous thromboembolic disease, dislocation, rigidity, spinal fluid leakage, neurological hurts, and others. ODI was evaluated after lumbar spine surgery.

#### Intervention

ERAS procedure is based on principles previously described by Kehlet [2, 3, 9, 27], focusing on standardization and evidence-based care in all parts of the treatment chain. In orthopedic surgery pathway, the changes needed in the current application of ERAS in the perioperative period can be summarized as follows [28, 29]: (1) surgery clinic: required surgery education class and specific identified care companion; (2) preoperative factors: oral multimodal analgesia, scopolamine patch, and short-acting spinal (preferred) or general anesthetic; (3) intraoperative factors: intravenous dexamethasone, 2 L of lactated Ringer’s, and tranexamic acid; and (4) postoperative factors: continuous adductor canal block for 48 h, physical therapy session on day of surgery, scheduled acetaminophen, NSAIDs, gabapentin, oxycodone PRN.

### Study quality assessment

The methodological index for non-randomized studies (MINORS) was used to assess the quality of the included studies [30, 31]. Sixteen items were scored as “0” (not reported), “1” (reported but inadequate), or “2” (reported and adequate). Two reviewers independently assessed the quality of the included studies.

### Data analysis and synthesis

Two reviewers independently extracted data, and the third reviewer checked the consistency between them. A standard data extracted form was used at this stage, including the authors, publishing date, country, sample size, age, gender, interventions, postoperative complications, readmission rate of patients after 30 days, postoperative Oswestry Disability Index (ODI), and 30-day mortality rate in the ERAS group and the non-ERAS group. For continuous outcomes, the mean, SD (standard deviation), and participant number will be extracted. For dichotomous outcomes, we extracted the total numbers and the numbers of events of both groups. The data in other forms was recalculated when possible to enable pooled analysis.

The data was extracted and input into the STATA software (version 12.0; StataCorp, College Station, TX, USA) for meta-analysis. A fixed effect model was applied when *I*^2^ < 50%, and a random effect model was applied when *I*^2^ > 50%. Odds ratio (OR) was calculated for dichotomous outcomes while weighted mean difference (WMD) for the continuous. Heterogeneity was assessed using the *x*^2^ and *I*^2^. We defined the acceptable heterogeneity by *P* value of *x*^2^ test > 0.10 and *I*^2^ < 50%. For heterogeneity data, subgroup analysis and sensitivity analysis were involved to detect the origin of heterogeneity and evaluate whether the other results would be markedly affected. Moreover, the effect of publication bias was investigated when the number of trials reporting the primary outcomes was 10 or more.

## Results

### Data retrieval

**Fig. 1:**
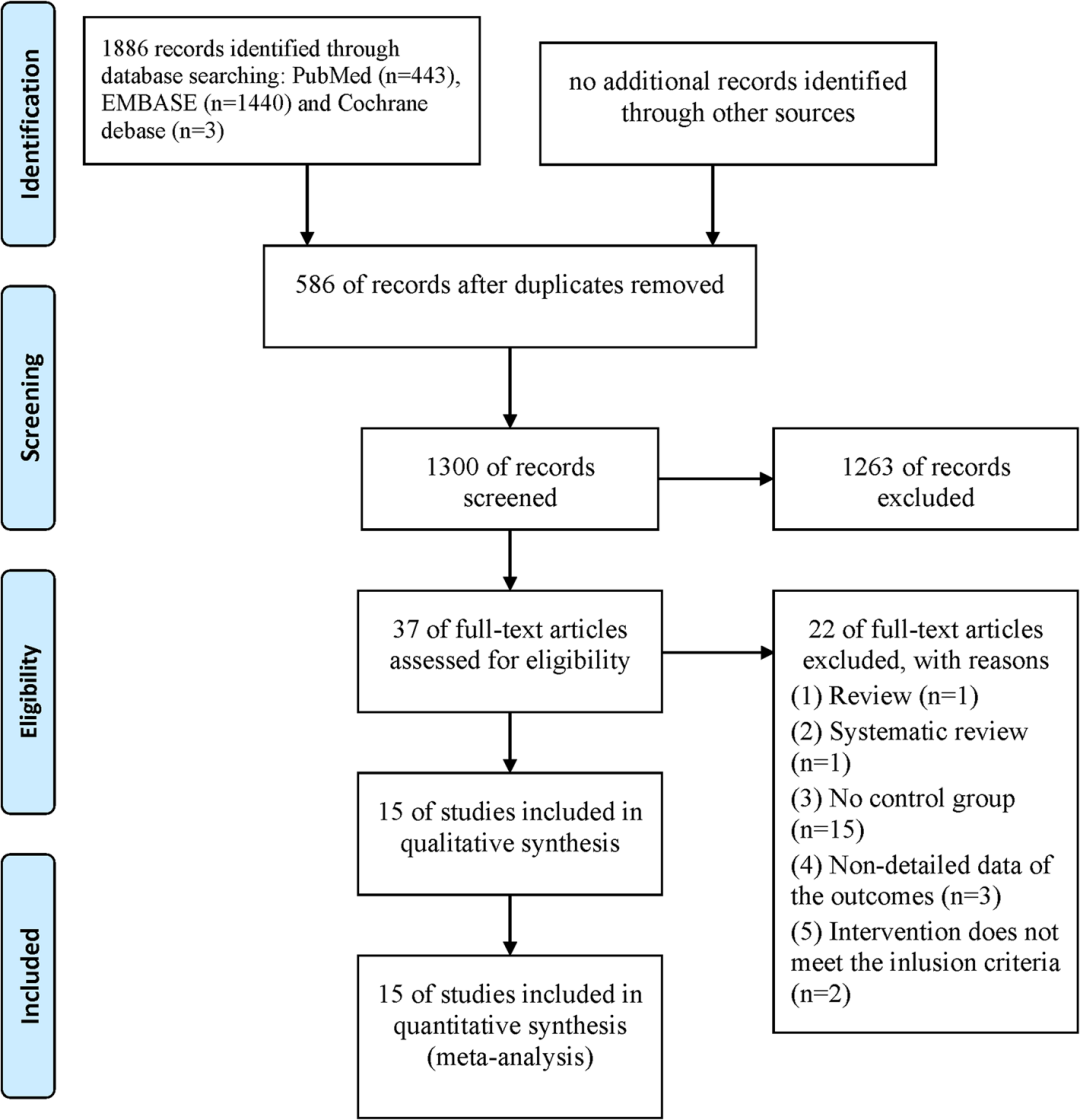
Flow of trials through the meta-analysis

In summary, a total of 1886 potential records were initially identified through PubMed, EMBASE, and Cochrane databases (detailed search strategies are reported in Additional file 2: Table S1). Based on our review of the title and abstract, 37 full-text papers were reviewed and 15 studies met the inclusion criteria. Twenty-two were excluded for reasons of “the papers were review or retrospective studies or from same investigation site” and some other reasons (details are shown in Fig. 1). Finally, 15 prospective and observational retrospective trials were included in this meta-analysis.

### Study and patient characteristics

The characteristics of all 15 included studies are summarized and shown in Table 1. The papers had similar distributions of sex, age, country, surgical site, and intervention, and all of them were published after 2008. The 15 studies involved 9700 participants who received ERAS and 11,143 who received a control intervention. Eleven [29, 32–41] had data on the incidence of postoperative complication, 6 papers [29, 32, 35, 37, 41, 42] reported 30-day readmission, 7 [24, 32, 35, 36, 39, 40, 43] referred to 30-day mortality rate, and ODI [40, 44] was recorded in 2 papers.

**Table 1:** The characteristics of the included studies

Source	Country	Surgical site	Intervention	No. of participants	Age, mean (SD)	Male/female
Auyong et al. [29]	USA	Joint	ERAS	126	66.2 (10.2)	44/82
			Traditional care	126	68.44 (9.98)	41/85
Christelis et al. [32]	Australia	Joint	ERAS	297	67 (10)	113/184
			Traditional care	412	68 (11)	164/248
den Hertog et al. [33]	Germany	Joint	ERAS	74	66.58 (8.21)	23/51
			Traditional care	73	68.25 (7.91)	20/53
Maempel et al. [34]	England	Joint	ERAS	84	69.8 (8.9)	42/42
			Traditional care	81	70.1 (10.5)	40/44
Malviya et al. [35]	England	Joint	ERAS	1500	68	711/789
			Traditional care	3000	69	1482/1518
McDonald et al. [36]	England	Joint	ERAS	1081	69 (11)	439/642
			Traditional care	735	70 (13)	307/428
Stambough et al. [42]	USA	Joint	ERAS	488	55 (19)	247/241
			Traditional care	281	59 (16)	126/155
Stowers et al. [37]	New Zealand	Joint	ERAS	100	66.7 (9.2)	47/53
			Traditional care	100	65.4 (12.5)	41/59
Khan et al. [43]	England	Joint	ERAS	3000	68 (10)	1390/1610
			Traditional care	3000	69 (10)	1482/1518
Pedersen et al. [38]	Denmark	Fracture	ERAS	178	82.2	42/136
			Traditional care	357	82.6	85/272
Eriksson et al. [24]	Sweden	Fracture	ERAS	80	85	65/15
			Traditional care	335	82	227/108
Liu et al. [41]	USA	Fracture	ERAS	2514	79.7 (11.7)	NA
			Traditional care	2488	79.3 (11.9)	NA
Macfie et al. [39]	England	Fracture	ERAS	117	82.5 (9.2)	89/28
			Traditional care	115	82.7 (8.7)	91/24
Wang et al. [8, 40]	USA	Spine	ERAS	38	65 (11)	17/21
			Traditional care	15	59 (12)	10/5
Nazarenko et al. [44]	Russia	Spine	ERAS	23	44.3	NA
			Traditional care	25	42.2	NA

*ERAS* enhanced recovery after surgery, *SD* standard deviation

### Quality assessment

The methodological quality assessment of the 15 included studies is summarized in Additional file 3: Table S2. Only two studies [40, 41] included mentioned both strengths and shortcomings, scoring as “2”; however, most of other studies provided an inadequate assessment. And no prospective calculation of the study size was found in our studies, scoring as “0.” Four studies [33, 34, 38, 40] had a minimum follow-up of 1 year, scoring as “2.” And the total scores ranged from 19 to 22 with a median value of 20, which means the high quality of the included studies. Publication bias assessment is described in Additional file 4: Figure S1, and there was no indication of a significant publication bias via funnel plot methodology.

### Meta-analysis

**Fig. 2:**
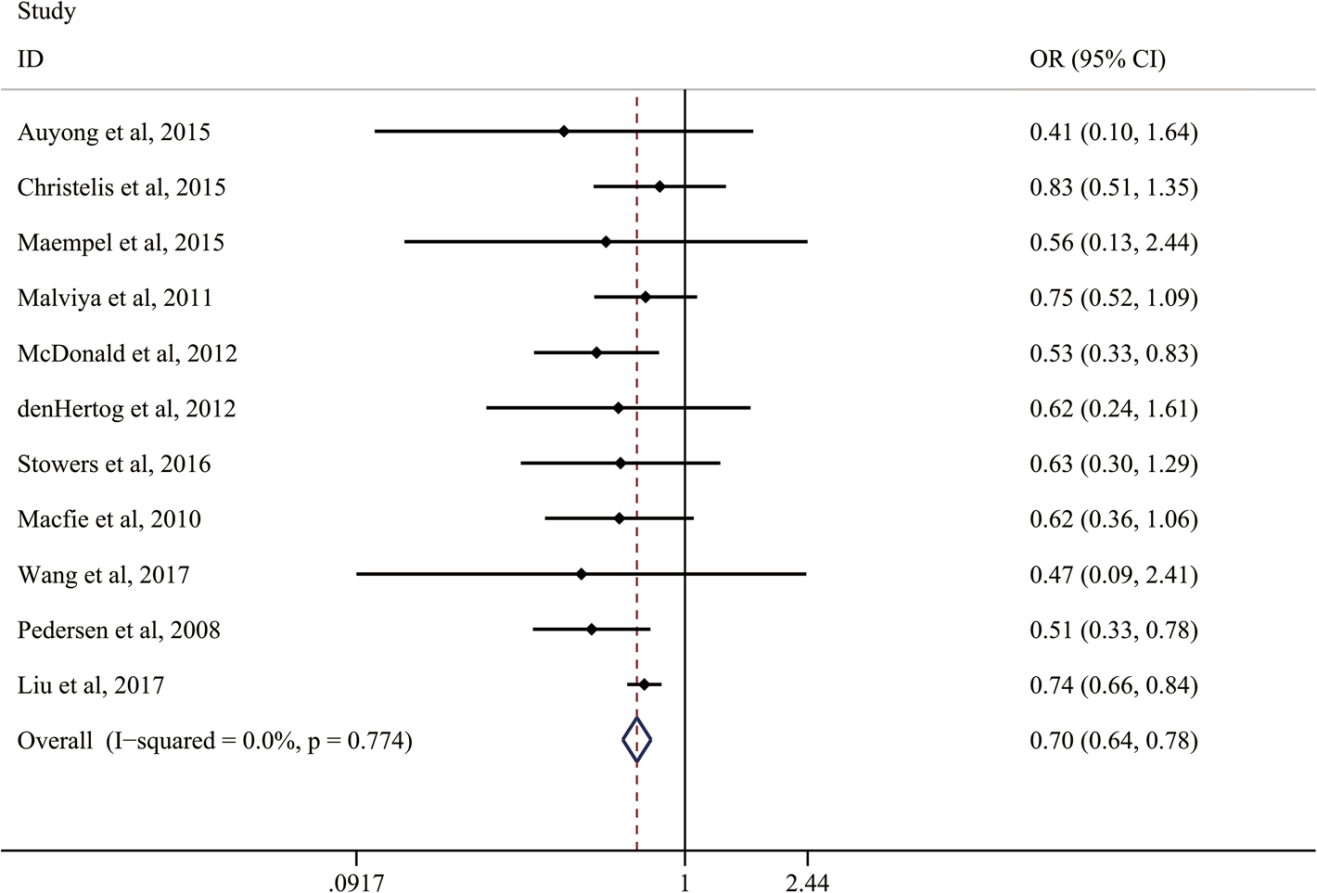
Comparison of the incidence of postoperative complications between the ERAS group and the control group

Eleven studies reported the data of the incidence of postoperative complication. There was no heterogeneity between the included studies (*I*^2^ = 0%, *P* = 0.774); fixed-effects model was adapted to analyze the results. The results of the meta-analysis are shown in Fig. 2, and the overall meta-analysis revealed that ERAS had a significantly lower incidence of postoperative complications than control groups (OR, 0.70; 95% CI, 0.64 to 0.78). Furthermore, the subgroup analysis of age, location, continents, and sample size showed no heterogeneity was found (all of the *P* > 0.10, Table 2). Additionally, sensitivity analysis was performed by excluding one trail in turn and recalculating the pooled OR for the remaining trials, which found that none of the studies affected the result (Additional file 5: Figure S2).

**Table 2:** Subgroup analysis of incidence of postoperative complications for each variable

Factors	Subgroups	Studies (*n*)	Patients (*n*) (experimental /control)	*I*^2^ (%)	Heterogeneity (*p*)	LOS, WMD (95% CI)
Average age	60–70 years	8	3300/4542	0	0.885	0.67 (0.54, 0.83)
	70–80 years	1	2514/2488	–	–	0.74 (0.66, 0.84)
	> 80 years	2	295/472	0	0.565	0.55 (0.39, 0.76)
Site	Joint	7	3262/4527	0	0.831	0.67 (0.54, 0.84)
	Fracture	3	2809/2960	36.4	0.208	0.72 (0.64, 0.80)
	Spine	1	38/15	–	–	0.47 (0.09, 2.41)
Continents	North America	3	2678/2629	0	0.612	0.74 (0.65, 0.84)
	Oceania	2	397/512	0	0.520	0.76 (0.51, 1.14)
	Europe	6	3034/4361	0	0.802	0.60 (0.49, 0.75)
Sample size	< 600	7	717/867	0	0.995	0.55 (0.42, 0.73)
	600–1000	1	297/412	–	–	0.83 (0.51, 1.35)
	> 1000	3	5095/6233	5.6	0.347	0.73 (0.65, 0.82)

*WMD* weighted mean difference, *CI* confidence intervals

**Fig. 3:**
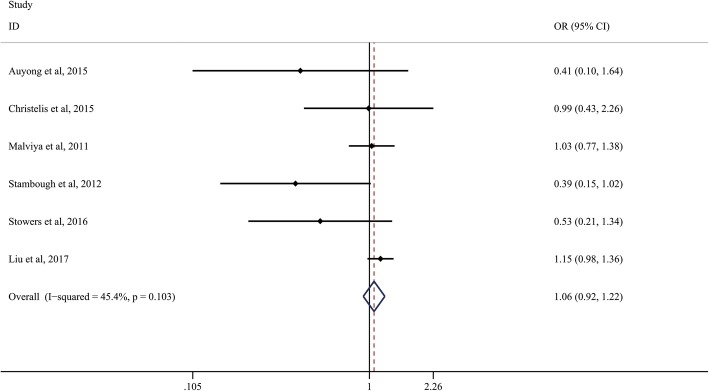
Comparison of 30-day readmission between the ERAS group and the control group

A total of six studies reported 30-day readmission between the ERAS group and control group. We applied a fixed-effects model to analyze the results since there was little heterogeneity between the included studies (*I*^2^ = 45.4%, *P* = 0.103). The results showed that no significant difference was found between both the ERAS and control groups in terms of 30-day readmission (OR, 1.06; 95% CI, 0.92 to 1.22, Fig. 3).

**Fig. 4:**
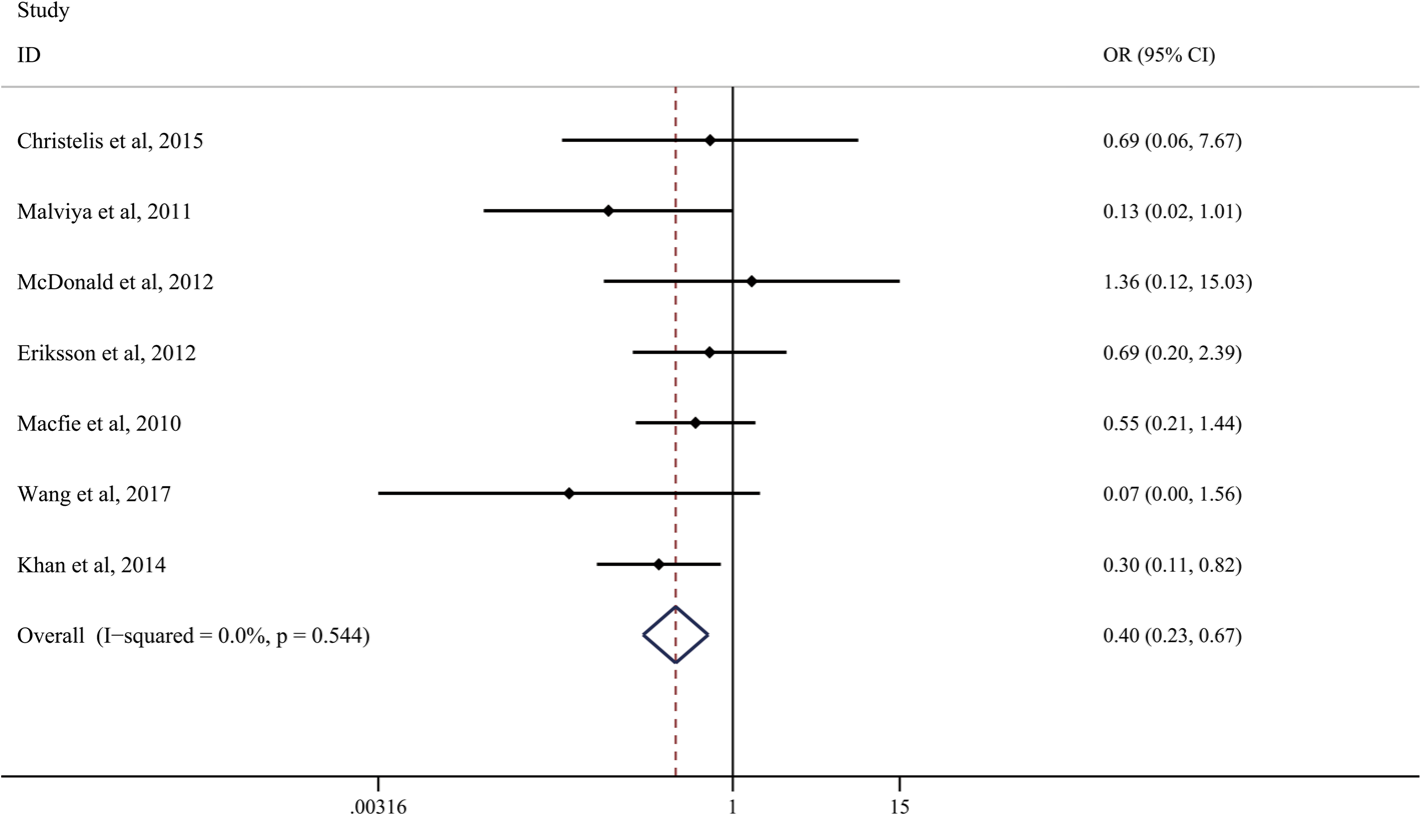
Comparison of the 30-day mortality rate between the ERAS group and the control group

A total of seven studies reported 30-day mortality rate between the ERAS group and control group. A fixed-effects model was applied to analyze the results since there was no heterogeneity between the included studies (*I*^2^ = 0.0%, *P* = 0.554). The pooled results indicated that the 30-day mortality rate in the ERAS groups was significantly lower than that in control groups (OR, 0.40; 95% CI, 0.23 to 0.67, Fig. 4).

**Fig. 5:**
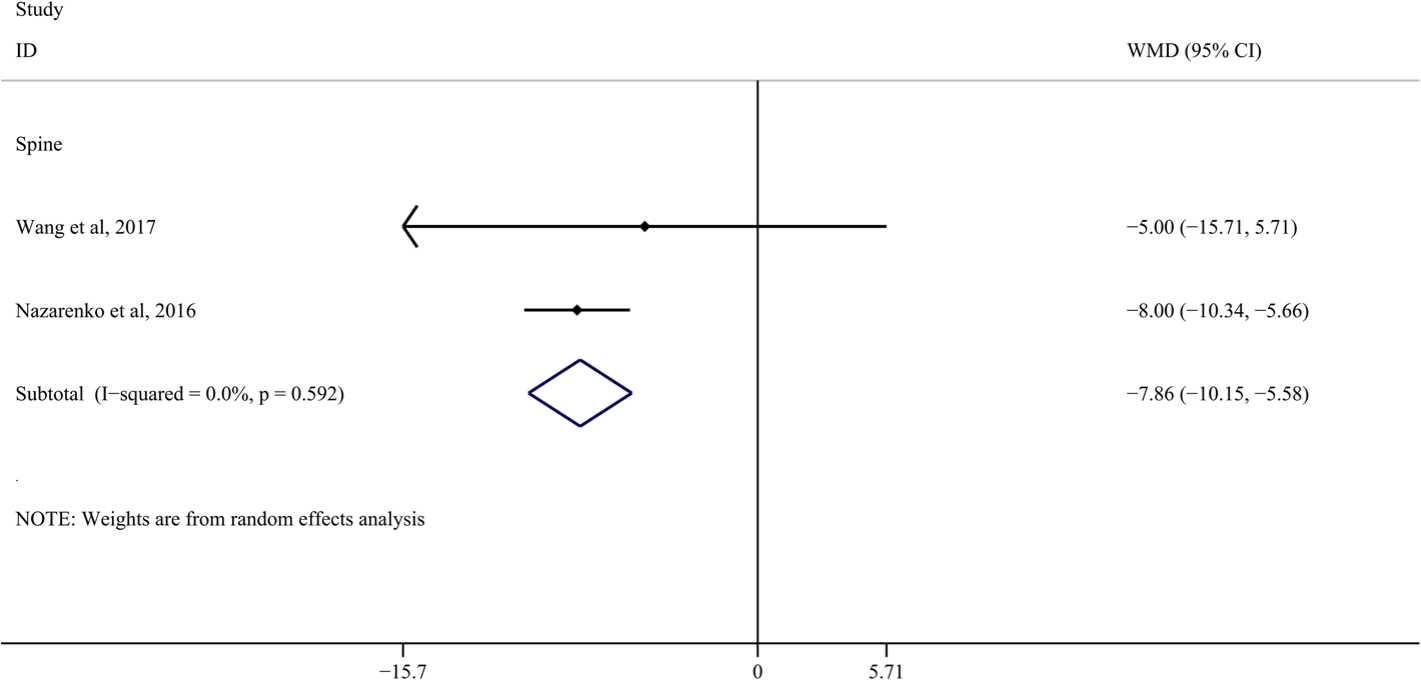
Comparison of Oswestry Disability Index (ODI) between the ERAS group and the control group

A total of two studies were available for ODI. The results suggested that ERAS significantly reduce the postoperative ODI (WMD, − 7.86; 95% CI, − 10.15 to − 5.58, Fig. 5). No obvious heterogeneity was observed; therefore, we used a fixed-effects model to pool the relevant data.

## Discussion

Since the introduction of ERAS concept by Kehlet in the 1990s, more and more studies have proved the safety and efficacy of this method in the perioperative period [45]. Although ERAS had reached a consensus in many surgical fields, there were few reports and systematic studies on ERAS in the fields of orthopedics because of their complex characteristics [8]. Some studies reported that ERAS was associated with better clinical outcomes such as LOS and incidence of postoperative complications [36, 38]. Conversely, for example, some reports held the opinion that ERAS did not appear to affect those in patients after orthopedic surgery [23, 29, 32, 34]. Therefore, the application of enhanced recovery after surgery in this field still remained a topic of debate.

For the majority of patients, the stress caused by the original injury will continue until the anesthesia was finished. And after anesthesia recovered, trauma caused by surgery may bring a greater degree of stress response and will remain in the whole process of functional exercise [46]. Meanwhile, orthopedic patients are subjected to greater anesthetic and surgical trauma, pain, hunger, high incidence of deep vein thrombosis, and other stimuli [45]. That is to say that the postoperative rehabilitation to improve the quality of life of patients is very critical. These characteristics determined that the accelerated rehabilitation model in orthopedic perioperative care had a wide-ranging applied background. Therefore, the concept of accelerated rehabilitation is worthy of attention, and it is also the future development trend and direction of perioperative care in orthopedics.

In this study, we analyzed the associations between ERAS and orthopedics using a meta-analysis to obtain a powerful conclusion. To the best of our knowledge, this is the first meta-analysis providing comprehensive insights into the efficacy and safety of the ERAS in orthopedics. Our meta-analysis showed that ERAS could reduce the incidence of postoperative complications, ODI, and 30-day mortality rate, but does not increase 30-day readmission rate without significant heterogeneity. Khan et al. [43] retrospectively analyzed 6000 patients who underwent TKA or THA. Similar to our results, they found that ERAS significantly reduced the incidence of postoperative complications such as myocardial infarction, readmission rate, and 30-day mortality rate compared with the traditional protocol. However, no significant differences were identified between the two groups regarding the incidence of stroke, gastrointestinal bleeding, pneumonia, deep vein thrombosis, and pulmonary embolism. In addition, Malviya et al. [35] evaluated 4500 consecutive unselected total hip replacements and total knee replacements. This large observational study showed the introduction of a multimodal enhanced recovery protocol had more advantages in reducing the incidence of postoperative complications but did not change the re-admission rate. The study also found that transfusion requirements were lower in the ERAS group than in the control group. What is more, Liu et al. [41] included a large-sample trial with 5002 patients undergoing orthopedic surgery and found that ERAS implementation was associated with reductions in hospital length of stay and postoperative complication rates, but not associated with the rate of 30-day readmission and hospital mortality. Therefore, consistent with our current results, most studies have shown that ERAS was a safe and effective program, which can speed up the patient’s recovery process, reduce their reliance on costly pain medication, and improve patients’ satisfaction. More importantly, the implementation of ERAS saves valuable medical resources.

However, the current meta-analysis still has several limitations. Firstly, most of them were not randomized controlled trials; the duration of follow-up was less than 5 years. Therefore, the level of evidence for this meta-analysis was not high. Secondly, for some outcomes in spine and trauma, we could only include a small number of studies in the analysis because we restricted the inclusion criteria of included studies which must contain control group so that several cross-sectional studies were excluded. And we may also ignore some confounding factors’ effects to our results [47, 48]. Thirdly, incomplete data recording was observed when we extracted clinical outcomes. Some functional outcomes were not performed due to the insufficiency of relevant data or high heterogeneity. Finally, definition of ERAS was different in different studies. Pooling of such data might lead to bias. Despite these weaknesses, our study can still provide some values for clinical reference.

## Conclusion

Our meta-analysis suggested that ERAS group had more advantages in reducing the incidence of postoperative complications, 30-day mortality rate, and ODI after orthopedic surgery, but not of 30-day readmission rate. Taken together, these results may support the routine use of ERAS in orthopedic surgery. However, further research with standardized, unbiased methods and larger sample sizes is needed for deeper analysis.

## Additional files

Additional file 1: Checklist. Preferred Reporting Items for Systematic Reviews and Meta-Analyses (PRISMA) (DOC 57 kb)

Additional file 2: **Table S1.** Search strategy (DOCX 13 kb)

Additional file 3: **Table S2.** Quality assessment of 15 included studies in MINORS (DOCX 16 kb)

Additional file 4: **Figure S1.** Analysis of publication bias for postoperative complications from 11 studies. (TIF 193 kb)

Additional file 5: **Figure S2.** Sensitivity analysis on the postoperative complications omitting individual studies one by one to evaluate whether the overall results could have been significantly influenced by one single study (TIFF 293 kb)

## Abbreviations

ERAS: Enhanced recovery after surgery
LOS: Hospital length of stay
ODI: Oswestry Disability Index
OR: Odds ratio scale
THA: Total hip arthroplasty
TKA: Total knee arthroplasty
WMD: Weighted mean difference
