# Supplementary material for: An enhanced recovery after surgery program in orthopedic surgery: a systematic review and meta-analysis
Source: J Orthop Surg Res. 2019 Mar 13;14:77. doi: 10.1186/s13018-019-1116-y (PMC6415350; doi:10.1186/s13018-019-1116-y)
Supplement: Supplementary file 2 — Table S1. Search strategy (DOCX 13 kb) [file 13018_2019_1116_MOESM2_ESM.docx]

| Additional file 2: **Table S1** Search Strategy | |
| --- | --- |
| **Database** | **Search strategy** |
| Pubmed | #1 "ERAS"[All Fields] OR "enhanced recovery"[All Fields] OR "fast track"[All Fields] OR "accelerated track"[All Fields]  #2 "orthopedics"[All Fields] OR "spine"[All Fields] OR "THA"[All Fields] OR "HHA"[All Fields] OR "TKA"[All Fields] OR "fracture"[All Fields] OR "hip"[All Fields] OR "knee"[All Fields] OR "arthroplasty"[All Fields] OR "joint"[All Fields]  #3 #1 and #2 |
