# Supplementary material for: An enhanced recovery after surgery program in orthopedic surgery: a systematic review and meta-analysis
Source: J Orthop Surg Res. 2019 Mar 13;14:77. doi: 10.1186/s13018-019-1116-y (PMC6415350; doi:10.1186/s13018-019-1116-y)
Supplement: Supplementary file 3 — Table S2. Quality assessment of 15 included studies in MINORS (DOCX 16 kb) [file 13018_2019_1116_MOESM3_ESM.docx]

Additional file 3: **Table S2** Quality assessment of fifteen included studies in MINORS.

| items  Authors | A | B | C | D | E | F | G | H | I | J | K | L | Total |
| --- | --- | --- | --- | --- | --- | --- | --- | --- | --- | --- | --- | --- | --- |
| Auyong et al | 2 | 2 | 2 | 2 | 1 | 1 | 2 | 0 | 2 | 2 | 2 | 2 | 20 |
| Christelis et al | 2 | 2 | 2 | 2 | 1 | 1 | 2 | 0 | 2 | 2 | 2 | 2 | 20 |
| denHertog et al | 2 | 2 | 2 | 2 | 0 | 2 | 2 | 0 | 2 | 2 | 2 | 2 | 20 |
| Maempel et al | 2 | 2 | 2 | 2 | 0 | 2 | 2 | 0 | 2 | 2 | 2 | 2 | 20 |
| Malviya et al | 2 | 2 | 2 | 2 | 0 | 1 | 2 | 0 | 2 | 2 | 2 | 2 | 19 |
| McDonald et al | 2 | 2 | 2 | 2 | 1 | 1 | 2 | 0 | 2 | 2 | 2 | 2 | 20 |
| Stambough et al | 2 | 2 | 2 | 2 | 0 | 1 | 2 | 0 | 2 | 2 | 2 | 2 | 19 |
| Stowers et al | 2 | 2 | 2 | 2 | 1 | 1 | 2 | 0 | 2 | 2 | 2 | 2 | 20 |
| Khan et al | 2 | 2 | 2 | 2 | 1 | 1 | 2 | 0 | 2 | 2 | 2 | 2 | 20 |
| Pedersen et al | 2 | 2 | 2 | 2 | 0 | 2 | 2 | 0 | 2 | 2 | 2 | 2 | 20 |
| Eriksson et al | 2 | 2 | 2 | 2 | 1 | 1 | 2 | 0 | 2 | 2 | 2 | 2 | 20 |
| Liu et al | 2 | 2 | 2 | 2 | 2 | 1 | 2 | 0 | 2 | 2 | 2 | 2 | 21 |
| Macfie et al | 2 | 2 | 2 | 2 | 1 | 1 | 2 | 0 | 2 | 2 | 2 | 2 | 20 |
| Wang et al | 2 | 2 | 2 | 2 | 2 | 2 | 2 | 0 | 2 | 2 | 2 | 2 | 22 |
| Nazarenko et al | 2 | 2 | 2 | 2 | 0 | 1 | 2 | 0 | 2 | 2 | 2 | 2 | 19 |

**Note:** A: A clearly stated aim; B: Inclusion of consecutive patients; C:Prospective collection of data; D: Endpoints appropriate to the aim of the study; E:Unbiased assessment of the study end point; F: Follow-up period appropriate to the aim of the study; G:Loss to follow-up less than 5%; H:Prospective calculation of the study size; I:An adequate control group; J:Contemporary groups; K:Baseline equivalence of groups; L:Adequate statistical analyses
