# Supplementary figures and images for: An enhanced recovery after surgery program in orthopedic surgery: a systematic review and meta-analysis
Source: J Orthop Surg Res. 2019 Mar 13;14:77. doi: 10.1186/s13018-019-1116-y (PMC6415350; doi:10.1186/s13018-019-1116-y)

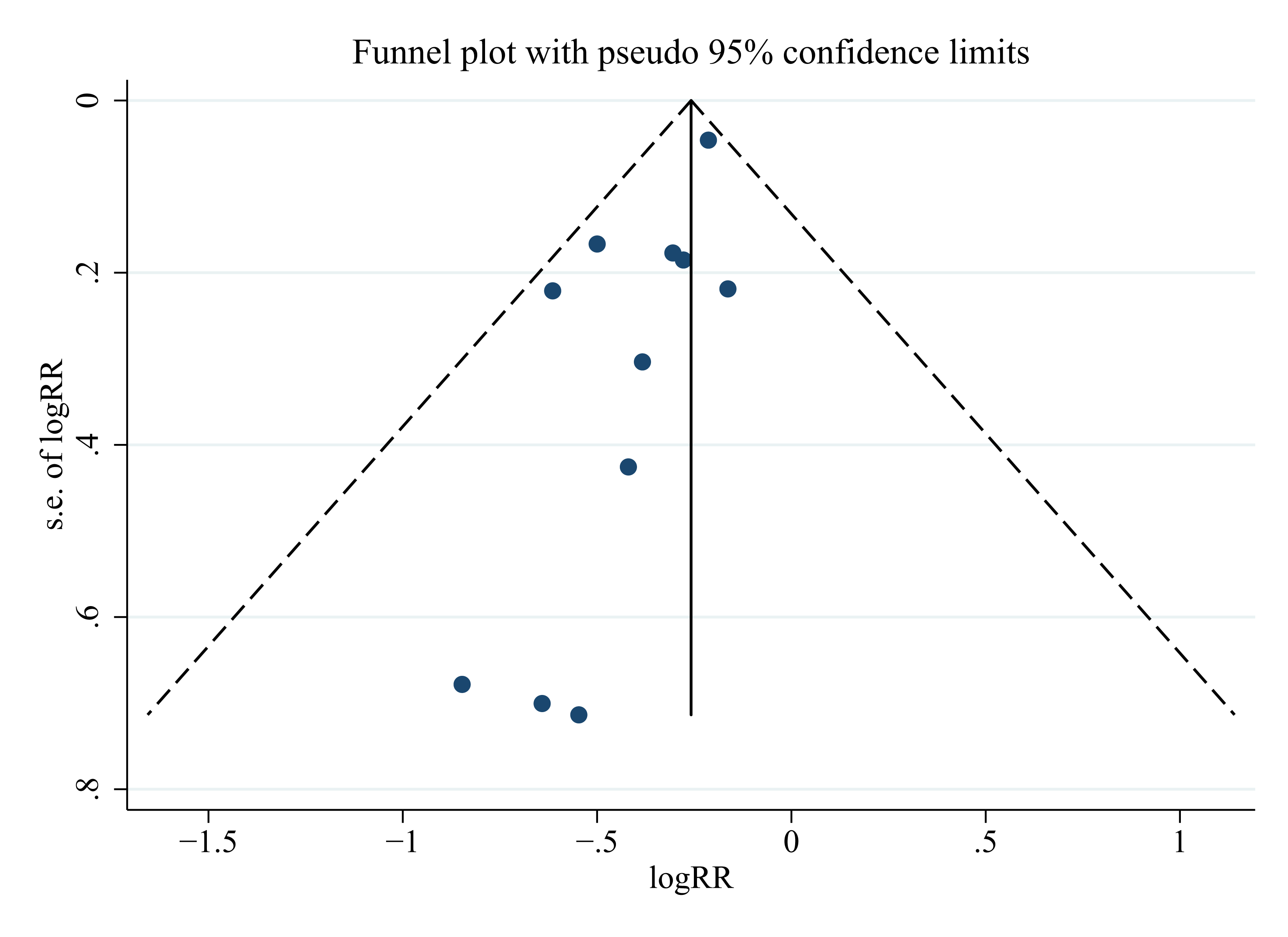

Supplement: Supplementary file 4 — Figure S1. Analysis of publication bias for postoperative complications from 11 studies. (TIF 193 kb) [file 13018_2019_1116_MOESM4_ESM.tif]
